# Metabolic and miRNA Profiling of *TMV* Infected Plants Reveals Biphasic Temporal Changes

**DOI:** 10.1371/journal.pone.0028466

**Published:** 2011-12-12

**Authors:** Ariel A. Bazzini, Carlos A. Manacorda, Takayuki Tohge, Gabriela Conti, Maria C. Rodriguez, Adriano Nunes-Nesi, Sofía Villanueva, Alisdair R. Fernie, Fernando Carrari, Sebastian Asurmendi

**Affiliations:** 1 Instituto de Biotecnología, CICVyA-INTA, Hurlingham, Buenos Aires, Argentina; 2 Consejo Nacional de Investigaciones Científicas y Técnicas (CONICET), Buenos Aires, Argentina; 3 Max Planck Institute for Molecular Plant Physiology, Wissenschaftspark Golm, Potsdam-Golm, Germany; Instituto de Biología Molecular y Celular de Plantas, Spain

## Abstract

Plant viral infections induce changes including gene expression and metabolic components. Identification of metabolites and microRNAs (miRNAs) differing in abundance along infection may provide a broad view of the pathways involved in signaling and defense that orchestrate and execute the response in plant-pathogen interactions. We used a systemic approach by applying both liquid and gas chromatography coupled to mass spectrometry to determine the relative level of metabolites across the viral infection, together with a miRs profiling using a micro-array based procedure. Systemic changes in metabolites were characterized by a biphasic response after infection. The first phase, detected at one dpi, evidenced the action of a systemic signal since no virus was detected systemically. Several of the metabolites increased at this stage were hormone-related. miRs profiling after infection also revealed a biphasic alteration, showing miRs alteration at 5 dpi where no virus was detected systemically and a late phase correlating with virus accumulation. Correlation analyses revealed a massive increase in the density of correlation networks after infection indicating a complex reprogramming of the regulatory pathways, either in response to the plant defense mechanism or to the virus infection itself. Our data propose the involvement of a systemic signaling on early miRs alteration.

## Introduction

Plants defend themselves against pathogens using a number of strategies and over the years much effort has been made to understand plant-pathogen interactions. The induction of genes and metabolites acting as defense or counter-defense compounds is a common feature that either enhances virus disease by suppressing host defense mechanisms or attenuate the infection orchestrating defense mechanisms [1], [2]. Furthermore, the emerging virus-host interaction picture suggests a highly complex network of plant responses and viral counter-responses which greatly impact in the plant physiology [3]. However, despite our broad knowledge concerning the infection effect at the whole plant level, documentation of the metabolic changes associated to this response remains scarce [4].

Systemic acquired resistance (SAR) and induced systemic resistance are enhanced states of broad-spectrum disease resistance in response to signaling and amplification processes triggered by pathogen infection [5], [6], [7], [8]. During these processes salicylic acid (SA), as methyl salicylate, and jasmonates (JA) have been proposed to play crucial roles during long-distance signaling [8], [9], [10], [11], [12]. It is well established that several other players are required to induce SAR such as hormones, lipids or proteins. However, how the systemic response is orchestrated still remains poorly characterized [13].

Plants produce an exceptionally large quantity of metabolites, which display a very broad chemical diversity [14], [15]. Thus, comparing metabolic profiles of infected plants versus their corresponding controls conceivably represents a powerful tool by which to unravel the biochemical pathways involved in multi-factorial disorders.

microRNAs (miRNAs) are small, endogenous RNAs that regulate gene expression in plants and animals by promoting cleavage or inhibiting translation of mRNAs coded by specific target genes [16], [17], [18], [19], [20]. miRNAs are involved in regulation of plant development, signal transduction, expression of transcription factors, protein degradation and interestingly are part of the response to biotic and abiotic stresses. We and others have demonstrated that after virus infection, miRs levels are altered and their alteration correlates with symptoms suggesting an important role of miRNAs in the manifestation of pathogen symptoms [21], [22], [23], [24]. It was also proposed that miRNAs function is exploited by the pathogens to regulate host gene expression for their own benefit [25], [26], [27], [28], [29], [30], [31], [32]. However, the underlying mechanisms of these effects are unclear. In plants, several groups have demonstrated that viral suppressors of post-transcriptional gene silencing (PTGS) interfere with miRNA-mediated regulation of host genes [28], [33], [34], [35], [36]. In the case of tobamoviruses, the replicase protein interferes with PTGS [37], [38], [39]. Work from our laboratory indicated that co-expression of the TMV movement and coat proteins in transgenic plants does not suppress PTGS, yet interferes with miRNAs accumulation [22]. Along the same line, it has been shown that *Turnip mosaic virus* (TuMV) induces bra-miR1885 accumulation resulting in cleavage of a TIR-NBS-LRR disease resistance target gene [40].

Here, we used a systemic approach by employing, both liquid chromatography (LC) and gas chromatography (GC), coupled to mass spectrometry (MS) to determine the relative levels of a large set of metabolites across the viral infection process, in parallel with an miRNA profiling using a micro-array based procedure. Identification of metabolites and miRNAs differing in abundance between control and infected samples may provide information concerning the pathways involved in signaling and defense which orchestrate and execute the response in plant–pathogen interactions. A clear biphasic response in both, metabolic and miRNA profiles was observed in accordance with the various stages of infection. Data obtained allowed us to search for correlation networks between both molecule types in response to the infection. These data thus suggest that there is an early systemic signaling stage independent of the virus's presence and a late second phase that correlates with virus accumulation.

## Results

### Experimental design

**Figure 1:**
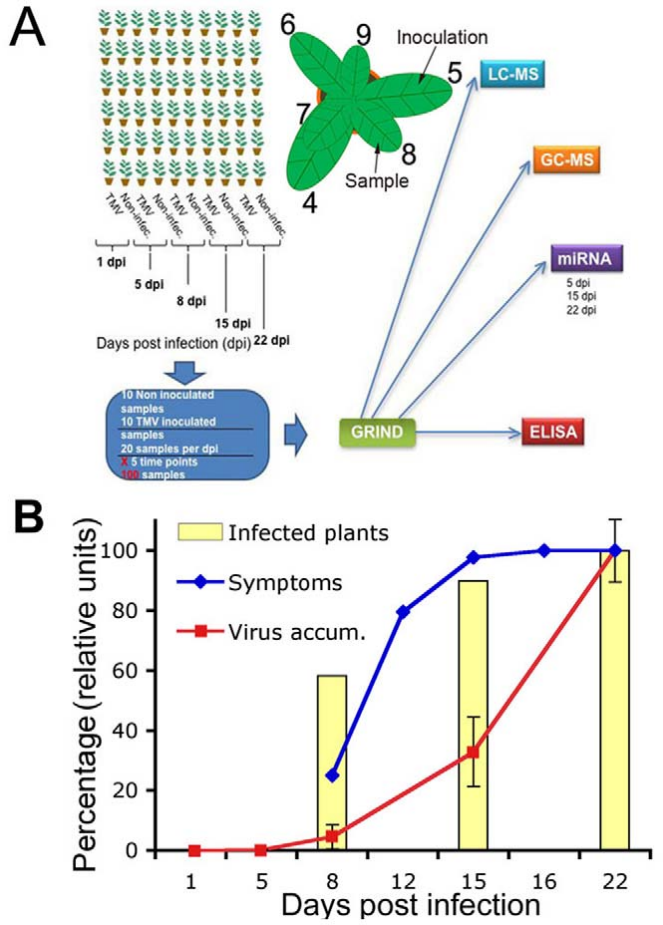
Experimental Design. **A**) Schematic representation of the experimental design. More than 100 five-week-old tobacco plants were inoculated with TMV or with mock solution in the fifth leaf. Third upper leaves (leaves number 8) were sampled at 1, 5, 8, 15 and 22 days post inoculation (dpi). Each sample was ground and the powder divided in 4 parts for virus detection by ELISA, miRNA detection by microarray and for primary and secondary metabolites detection by GC-MS and LC-MS respectively. **B**) TMV infection progression along the experiment in virus-inoculated plants. Bar graph: percentage of TMV-inoculated plants systemically accumulating virus as detected by ELISA in the sampled leaf along the infection. Squares line: Relative systemic accumulation of viral CP quantified by ELISA assays for samples at each time point. Diamonds line: percentage of TMV-inoculated plants showing characteristic symptoms under naked eye inspection. n = 10 plants per each time point.

To establish differences in metabolites and miRNAs accumulation occurring during plant-virus compatible infections, the pathosystem selected in the present study consisted of *N. tabacum* plants (Xhanti, nn) grown under greenhouse controlled conditions and infected with *Tobacco mosaic virus* (TMV). The fifth leaf from five-week-old plants were either TMV- or mock-inoculated. The third leaves above from the inoculated one (leaf number eight) were sampled at one, five, eight, 15 and 22 days post-inoculation (dpi) (10 plant/treatment and time point) (Figure 1A). Viral accumulation was followed by an ELISA assay detecting the TMV coat protein (CP) in the sampled leaves (Figure 1B). Non-detectable virus was found at either one or five dpi. At eight dpi the percentage of plants with virus accumulation in the sampled leaf reached 60%; however CP accumulation levels were very low (4% of the maximum level reached at 22 dpi) at this stage. The CP levels increased rapidly at 15 dpi (32% of the maximum level reached at 22 dpi), reaching the maximum at 22 dpi (Figure 1B). Symptoms were first noticed at eight dpi in only 20% of the plants with very mild mosaic in leaves. At 22 dpi all the inoculated plants showed characteristically severe TMV symptoms (Supplemental Figure S1).

### Viral infections produce a systemic biphasic alteration on leaf metabolism

**Figure 2:**
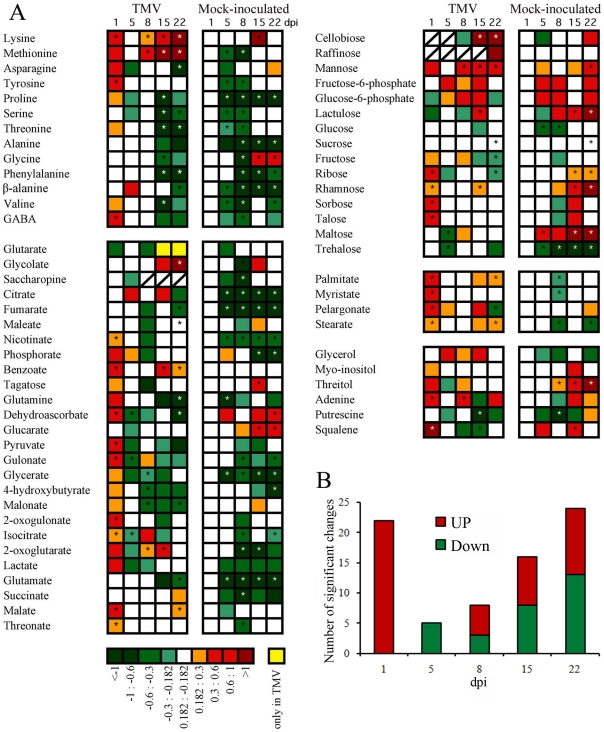
Relative metabolite content of TMV infected plants detected by GC-MS. **A**) Heat map representing changes in relative metabolite contents of TMV-inoculated and control plants detected by GC-MS experiments. The average accumulation of each metabolite was calculated in each time point and treatment (n≥6, per time/treatment). TMV panel shows the average metabolites level ratios calculated between TMV- and mock-inoculated plants (TMV panel) for each time point. Mock-inoculated panel shows the relative average metabolites logarithmic ratios with respect to 1 dpi data at different time points. Asterisks indicate statistically significant differences (*P*<0.01) by Student's *t* test. **B**) Number of metabolites with significant changes along TMV infection extracted from figure 2A (TMV panel). Dpi = days post inoculation.

**Figure 3:**
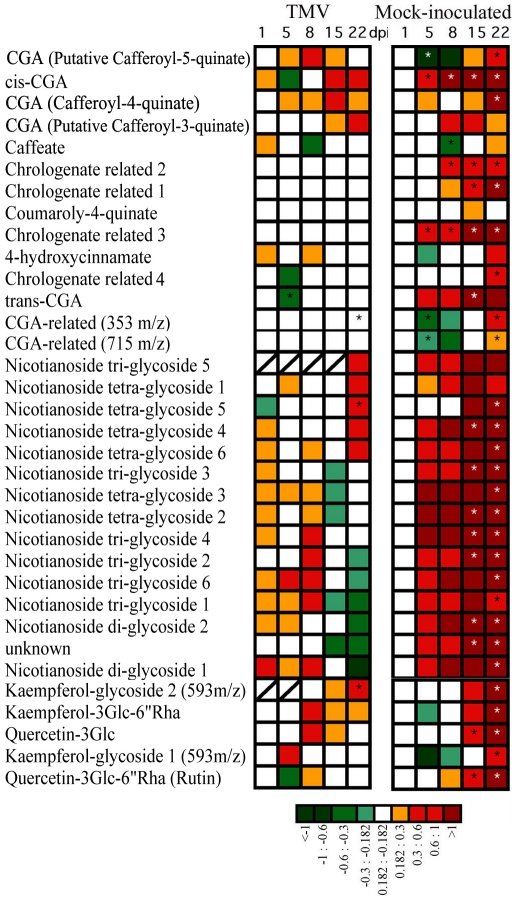
Relative metabolite content of TMV infected plants detected by LC-MS. Heat map representing changes in relative metabolite contents of TMV-inoculated and control plants detected by LC-MS experiments. On the TMV panel the logarithmic ratio between the levels of each metabolite in TMV-inoculated versus the mock-inoculated plants for each time point are shown (similar to Figure 2). On the mock-inoculated panel, the ratios were calculated between data of each time point of mock-inoculated samples versus the first dpi of mock-inoculated (similar to Figure 2). Asterisks indicate statistically significant differences (*P*<0.01) by Student's *t* test.

GC- and LC-MS analyses were both performed with the aim of detecting a relatively comprehensive overview of metabolic alterations following viral infection. The relative levels of 64 primary (GC-MS) and 34 secondary (LC-MS) metabolites were analyzed in split samples obtained from the same TMV- and mock-inoculated plants across the 22 day period (Figures 1, 2 and 3). Figure 2A (see Supplemental Figure S2A for detailed data) shows primary metabolite changes detected in systemic leaves from TMV- and mock-inoculated plants. The TMV panel (left side) of Figure 2A shows the logarithmic ratio between the levels of each metabolite in TMV- versus mock-inoculated plants at each time point whereas the mock-inoculated panel (right side) shows the ratio between data of each time point of mock-inoculated plants versus the first dpi of mock-inoculated. These two relative values indicated the metabolic alteration produced by the virus infection at each time point (TMV panel) and the metabolic changes produced as a consequence of the development along the time of the experiment (mock-inoculated panel). The virus-mediated metabolic alterations showed two temporal phases distinct in both the trend and number of altered metabolites (Figure 2AB, TMV panel). An early first phase (one dpi) was mainly characterized by a rapid and significant increment in the level of a number of metabolites whilst the second phase, at later stages of infection (specifically at 15 and 22 dpi), revealed both increases and decreases in metabolite levels. The early phase spanned the first 24 hours of infection when viral accumulation was undetectable in the sampled leaf (Figures 1B and 2). By contrast, in the second phase the number of statistically significant changes correlated with the increasing accumulation of the virus along the infection progress (Figures 1B and 2B).

In the first stage there were three groups of metabolites that are of high interest from the host-pathogen interaction perspective: ascorbate pathway-related metabolites, fatty acids and phenolic compounds (Figure 2A). For the ascorbate acid pathway dehydroascorbate, gulonate and 2-oxo-gulonate were altered (for details see Supplemental Figure S2A). Several of the detected altered fatty acids (palmitate, myristate, pelargonate and stearate) are precursors of the JA biosynthetic pathway and all of them co-ordinately increased at this first phase. Similarly, regarding phenolic compounds, one of the salicylate precursors, benzoate, also showed significantly increased levels both at very early (one dpi) and later (15 and 22 dpi) stages. In addition, γ-amino butyric acid (GABA) is another stress-related molecule which was statistically increased during this early phase. Moreover, a group of sugars (ribose, rhamnose, and sorbose), and several organic acids associated with the tricarboxylic acid cycle (TCA) pathway (malate, isocitrate and pyruvate), displayed a rapid increase in their contents. At this early time point secondary metabolites were not significantly altered (Figure 3 TMV, left panel).

Despite the clear metabolic alterations observed in systemic leaves after 24 hours of infection, four days later only a few metabolites displayed significant alterations in their contents (five dpi). Higher numbers of statistically significant changes in metabolite levels were, however, observed at eight dpi and especially at 15 and 22 dpi (Figure 2B). This second phase of metabolic changes strongly correlated with the level of virus accumulation (Figures 1B and 2B). The most altered metabolite groups in this phase were amino acids, since more than half of them showed significant changes at 15 and 22 dpi. However, there were also changes in fatty acids levels which may be related to the disruption or production of endomembrane components which is known to occur during virus replication, movement and spread [41], [42]. In contrast to the above described metabolic changes detected in systemic tissues following TMV infection, the heat maps shown in Figures 2A and 3, mock-inoculated panels (right side) dissected metabolic changes during leaf development along the experiment. Figures 2A and 3, mock-inoculated panels displayed metabolite levels as logarithmic ratios between one dpi and each of the subsequent time points sampled. The TCA-related metabolites (citrate, fumarate, isocitrate, 2-oxoglutarate and succinate (see Supplemental Figure S2B) as well as several amino acids such as proline, alanine, phenylalanine and valine were strongly reduced in older leaves. Moreover, massive inductions in secondary metabolites accumulation such as, chlorogenate and flavonoid-related metabolites and nicotianoside related compounds were registered upon aging of the leaves (Figure 3 and Supplemental Figure S2B).

It is known that as tobacco leaves become older they increase resistance to viral infection in comparison to younger leaves. Chlorogenate and flavonoid compounds have previously been reported to confer pathogen resistance [43], [44], hence it is possible that the elevated natural resistance on development is due to the increased accumulation of those metabolites. However, neither flavonoids nor chlorogenate compounds were induced by the infection (Figure 3) suggesting that these metabolites do not belong to inducible defence pathways in accordance with results reported by Maher et al [43], similar behaviour were shown by the large number of nicotianosides detected.

### Virus accumulation correlates with amino acid depletion in leaves

**Figure 4:**
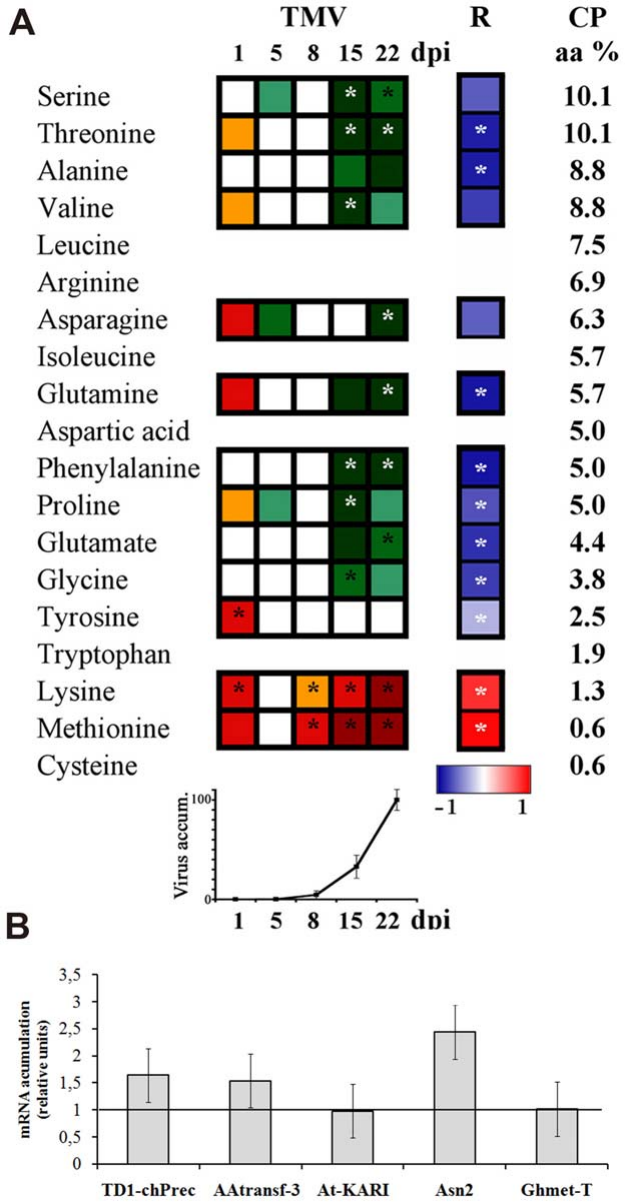
Relative abundance of free amino acids in TMV infected Plants. **A**) Changes in the relative abundance of free amino acids between TMV-inoculated and control plants detected by GC-MS experiments (same data as Figure 2). Pearson correlation between CP and free amino acids levels was calculated and the correlation coefficients R are showed on a blue-red false color scale. Asterisks indicate statistically significant correlations (*P*<0.01). Amino acid composition of CP is listed as a percentage of abundance (aa %) and CP accumulation during virus infection is shown as line graphic. **B**) Relative mRNA accumulation of genes involved in amino acids synthesis detected by qPCR: TD1-chPrec = chloroplast threonine deaminase 1 precursor, AAAtransf3 = Branched-Chain Amino Acid Aminotransferase 3, AtKARI = similar to *A. thaliana* ketol-acid reductoisomerase, Asn2 = Asparagine Synthetase 2, GHmetT = Glycine Hydroxymethyltransferase.

As mentioned above several amino acids showed a marked reduction in their contents particularly at later stages of infection (15 and 22 dpi) (Figure 2A, TMV panel). Moreover, free amino acids residues present on the TMV CP decreased significantly in the infected plants at later stages (Figure 4A). More precisely, negative correlations between free amino acid contents in the TMV-inoculated plants and the accumulation of virus CP were found on most of the amino acids that compose CP (Figure 4A, see R panel). Furthermore, when a second correlation was calculated between the mentioned correlation coefficients (amount of CP and amino acid level) and the percentages of each amino acid on the CP composition (Figure 4A) an R = −0.82 with a p-value of 0.002 was obtained, indicating that the patterns of amino acid changes also strongly correlated with the frequency of these amino acids in the TMV CP composition. These results suggested that the reduction of free amino acids is a consequence of the massive CP production.

In order to investigate whether the decrease of these amino acids correlated with the expression of their biosynthetic pathway genes, the steady-state mRNA levels of a chloroplast threonine deaminase 1 precursor (TD1-chPrec), branched-chain aminotransferase (AAtransf 3), a ketol-acid reductoisomerase (KARI), asparagine synthase 2 (ASN2) and glycine hydroxymethyltransferase (GhMetT), were measured by RT-qPCR at 22 dpi (Figure 4B). Those genes were selected based on *Arabidopsis* amino acids pathways followed by a search for orthologous genes in tobacco data bases to obtain the tobacco sequences. None of the genes analysed displayed statistically significant differences in their mRNA accumulation patterns when comparing TMV-infected versus mock-inoculated leaves. These results suggest that the observed reduction in free amino acids accumulation may be mostly a consequence of an increased use of these pools of amino acids during the massive CP production (Figure 4 A) rather than changes in the rates of their biosynthesis.

### Biphasic alteration of miRNAs levels during TMV infection

**Figure 5:**
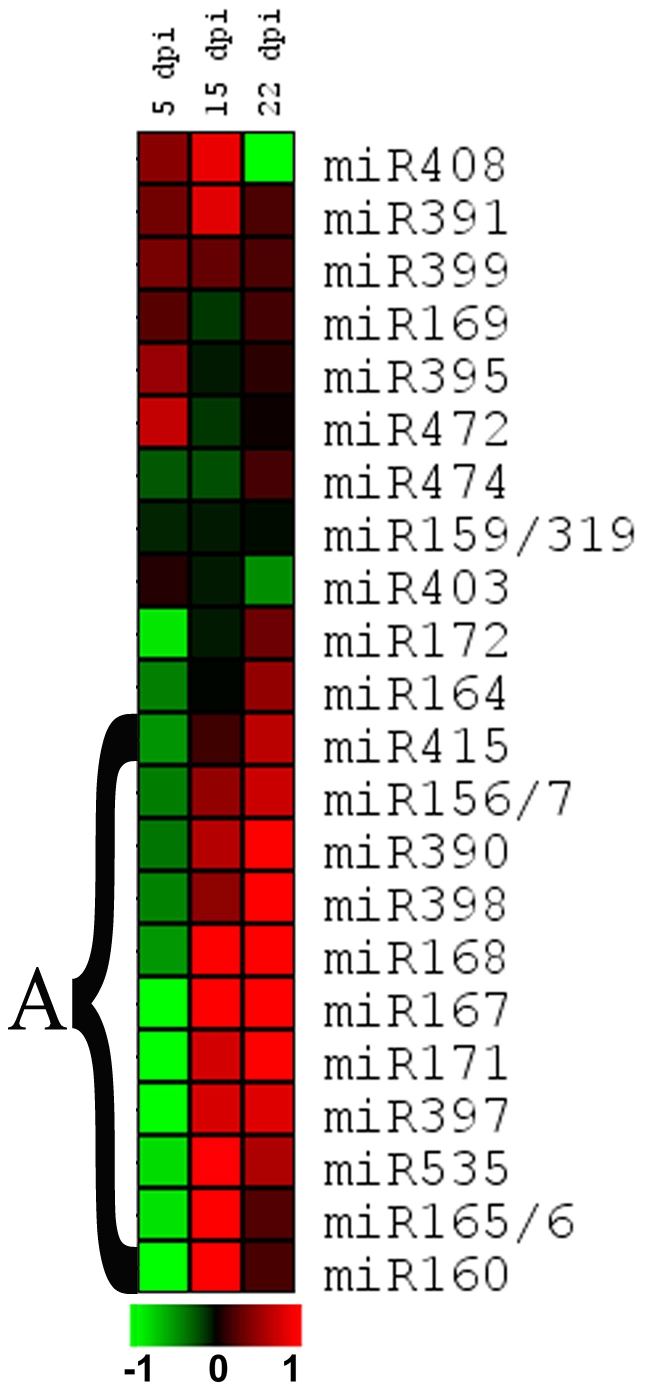
Temporal profiling of miRNAs accumulation following TMV infection. Systemic, non-inoculated tobacco leaves were sampled from virus- and mock-inoculated plants (n = 6 for each day and treatment) at 5, 15 and 22 dpi. miRNAs accumulation in TMV-inoculated plants were compared to those corresponding to mock-inoculated plants and the relative accumulation was calculated as the ratio between virus- and mock-inoculated samples at each time point. These ratios were log2 transformed and further analyzed with hierarchical clustering with average linkage. False-color scale indicates lower (green) or greater (red) miRNA accumulation in TMV-infected plants respect to mock-inoculated ones. The horizontal bar shows the log2-based scale. See Materials and Methods for further details.

To investigate the level of miRNA changes during TMV infection in the exact same samples for the metabolic and ELISA studies (Figure 1A), the accumulation of several miRNAs was measured by using a microArray containing all the known probes for detection of plant miRNAs present in the miRbase release 9 [45]. Small RNAs were extracted from the samples taken at five, 15 and 22 dpi from the TMV- and mock-inoculated plants (Figure 1A). The studies revealed a temporal miRNAs level alteration after the infection unexpectedly showing two clear distinct stages. At five dpi when no virus was detected (Figure 1B and Supplemental Figure S3), several of the analyzed miRNAs were down-regulated compared with mock-inoculated plants (Figure 5 and Supplemental Figure S4) while most of the miRNAs were up regulated at 15 and 22 dpi, as expected, when viral accumulation was high on the sampled leaf. In particular, miR415, mir156/7, mir390, miR398, miR168, miR167, miR171, miR397, miR535, miR165/6 and miR160 form a cluster (named cluster A) that was down- and up-regulated at the early (5 dpi) and later (15 and 22 dpi) stages of infection respectively, when compared with mock-inoculated plants. Within this group miR165/6 and miR160 even thought behave as the other at 5 and 15 dpi show smaller up regulation at 22 dpi. The miRNAs miR172 and miR164, showed a similar tendency considering 5 and 22 dpi. It is noticeably group A mentioned miRNAs are described as responsive to different biotic and abiotic stresses [46], [47], [48]. Also, these groups show a biphasic alteration similar to that observed for the leaf metabolome after the infection. There are another set of nine miRNAs out of twenty two that also showed changes but without showing this biphasic trend. Finally it worth to mention that miR403 and in stronger manner miR408 were the two unique miRNAs down regulated at late stage (22 dpi) probably indicating a different alteration mechanism.

### miRNAs and miRNA-targets accumulation during two different tobamovirus infections at early time points

In order to provide a validation of the microArray data we measured a set of miRNAs and also some of their mRNA target levels on an independent experiment by means of RT-qPCR. To pursue this analysis a new infection assay was performed on tobacco plants using two viruses, TMV and *Sunn-hemp mosaic virus* (ShMV), these two viruses differ markedly on the symptoms they produce on tobacco; severe and mild respectively (see Supplemental Figure S1 for a disease severity comparison). The inoculation and leaf sampling followed same scheme as the previous assay (Figure 1A) but the level of inoculums was reduced (dilution 1/10). The aim of use reduced inoculums was to produce a longer early phase by means of slowing down the initial step of the infection, in agreement with this idea the sampling time was performed at 6 dpi and 11 dpi. To show this effect (extended early phase) the level of TMV-CP of samples at 6 and 11 dpi was analyzed and compared to 22 dpi by means of RT-qPCR assay. The Supplemental Figure S5 show a ratio of CP level between infected and non infected plants for each time point; only 11 dpi showed a statistically difference, and at this time point the TMV-CP accumulation was less than 1% of the maximum level detected at 22 dpi (Supplemental Figure S5) indicating that at 11 dpi the sampled leaf is at the early stages of virus accumulation.

**Figure 6:**
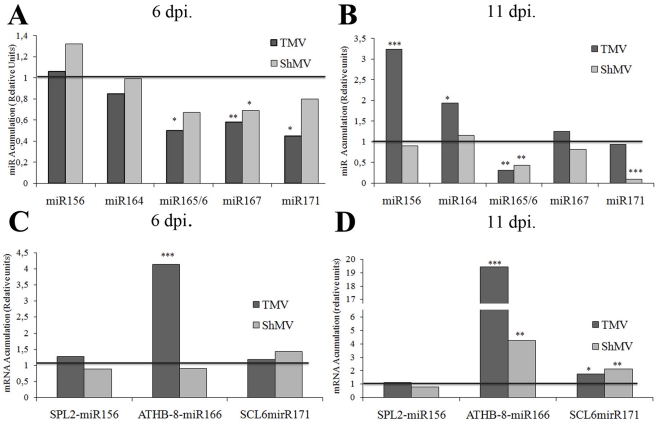
TMV and SHMV infected plants miRNAs and mRNA targets accumulation levels at early time points by qPCR. Relative accumulation of miRNAs 156, 164, 165/6, 167 and 171 (**A,B**), and miRNAs 156, 166, and 171 target genes (**C,D**) on virus-infected plants with **low inoculum**. A ratio between each virus- and mock-inoculated plants is shown at 6 days post inoculation (dpi) (**A,C**) and 11 dpi (**B,D**). * means p-value<0.1; ** means p-value<0.05 and *** means p-value<0.01.

At 6 dpi both virus infections produced a reduction of miRNA levels (Figure 6 A) of miRNAs belonging to Cluster A (miR 165/6, miR167 and mir171) confirming the microarray results at early time point. On this work we focused on miRNAs of cluster A since those miRNAs possess established link with stress response and were clear exponents of the biphasic alteration. Comparing both infections, the most severe virus (TMV) seems to produce faster effects. To determine the activity of those miRNA changes, the levels of their targets were quantified in the same samples. Even though quite recently Frazier et al [49] reported miRNAs sequences, gene structure prediction and same miRNAs sequence validation in tobacco, scarcely data about miRNA target was available for tobacco, so it was first required to select and validate tobacco miRNA target mRNAs. We were able to find and validated three of target genes by RNA ligase-mediated rapid amplification of cDNA ends (RLM-RACE) (Supplemental Figure S6). The target genes were: a miR156-regulated Squamosa promoter binding protein-like 2 (SPL2), a miR165/166-regulated Homeobox gene 8 (ATHB-8) and a miR170/171-regulated Scarecrow-like transcription factor 6 (SCL6). Interestingly, at 6 dpi ATHB-8 mRNA negatively correlated with miR166 levels in the TMV (most severe)-infected plants but not in the mild ShMV virus (Figure 6C).

At 11 dpi in TMV-infected plants, miR156 and miR164 were up-regulated in a statistically significant manner (Figure 6B) evidencing the transition from the first to the second phase, where most of the miRNAs accumulate to higher levels (Figure 5). On the other hand, ShMV-infected plants showed an overall reduction in miRNAs accumulation levels, being miR165/166 and miR171 of statistical significance. These results suggested that miRNAs and target genes alteration might be delayed in the plants infected with the less severe virus. At 11 dpi, ShMV and TMV-infected plants showed a statistically significant increase of miR165/6 and miR171 targets (Figure 6D). Furthermore, the up-regulation of miR156 in TMV-infected plants at 11 dpi did not produce the expected alteration on SPL2 mRNA at the same infection stage. This latter behavior may indicate a reduction on the miRNA activity together with its increased accumulation levels, probably due to the virus PTGS suppressor activity.

### Pre-miR166 accumulation is altered upon virus infection

**Figure 7:**
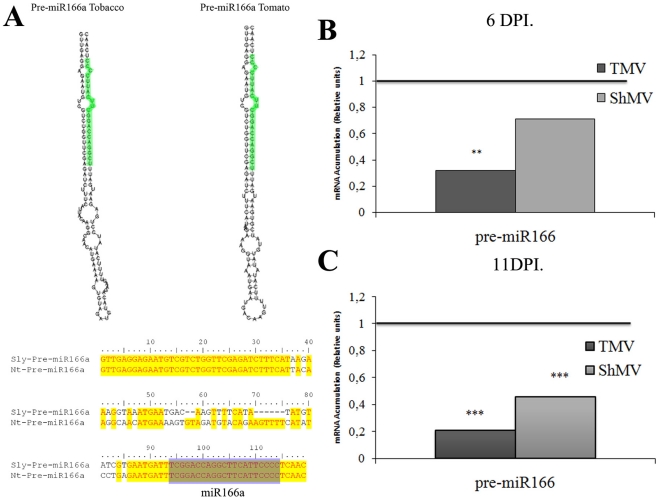
Pre-miRNA166 is reduced after virus infection. **A**) Predicted fold-back secondary structures of tobacco and tomato miRNA166a precursors as determined by the *RNAfold* program. Mature miRNA sequences are highlighted in green. Clustal alignment between Pre-miRNA166a sequences from tobacco and tomato. (**BC**) Pre-miRNA166a qPCR quantification in plants infected with TMV or ShMV, (**B**) 6 dpi and (**C**) 11 dpi. ** means p-value<0,05 and *** means p-value<0,01.

In order to analyze whether the observed alteration on miRNAs levels at early stages of infection had a transcriptional component as observed on an earlier work at late stages of infection in *Arabidopsis* [21], we decided to analyze the accumulation of immature precursors of miRNAs (pre-miRNAs) by qPCR in the same samples used before (Figure 6). In spite of our effort to identify the pre-miRNAs sequence for each detected mature miRNA using the tomato (*Solanum lycopersicum*) and *Arabidopsis* orthologous genes sequences, we were only able to PCR-amplify pre-miR166a. Figure 7A shows the comparisons of sequences and the predicted structure of the tomato pre-miR166a and the tobacco fragment isolated. At 6 dpi, only the TMV-infected plants presented significant lower level of pre-miR166a compared to the mock-inoculated plants (Figure 7B). However at 11 dpi both viruses produced reductions on pre-miR166a levels (Figure 7C), in agreement with the reduced accumulation of the mature miRNAs observed, suggesting a possible transcriptional down-regulation mechanism.

These data all together suggest that in the absence of detectable virus in systemic leaves (6 dpi), miR166 and pre-miR166a accumulation decrease and its target mRNA increase following the infection in the TMV-infected plants (Figures 6A and C and 7B). However, at 11 dpi the amount of pre-miRNA is reduced in plants infected with both virus (Figure 7C) showing the expected increase of ATHB-8 target mRNA level (Figure 6D). In conclusion, in agreement with the levels of symptoms both virus produced on tobacco, the most severe virus (TMV) altered miRNA accumulation and activity more rapidly than ShMV did.

### A massive increase in the density of correlation networks indicates a complex reprogramming of the regulatory pathways triggered by viral infection

**Figure 8:**
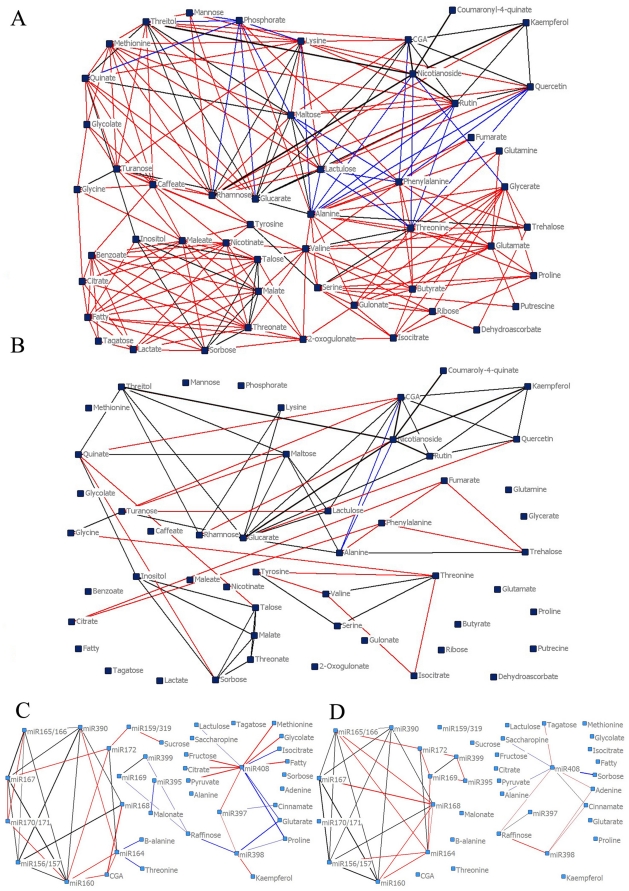
Non-parametric Spearman's correlation analysis. Metabolites-metabolite (AB) or miRNA-metabolites level (CD) correlation was calculated between inoculated plants (AC) or mock-inoculated plants (BD). Significant correlation (*p* = 0.001, for AB and *p* = 0.01 for CD; R>0.5) between two metabolites was drawn with color lines. Positive correlations were drawn with red lines, negative with blue lines. Correlations present in both TMV inoculated (AC) and mock-inoculated plants (BD) were drawn in black, regardless the sense of the correlation.

In order to assess the integrated behavior of the metabolic network during virus infection, a combinatorial analysis of metabolites was run by non-parametric Spearman's rank-order correlation analysis, with the significance threshold set at *p*<0.001. The resultant matrix revealed a dense correlation network in infected plants comprising 249 significant connections (Figure 8A). In contrast the same analysis in mock-inoculated samples rendered a network of only 69 significant connections (Figure 8B). In order to reduce the complexity of the figure, all the detected fatty acid (fatty), all detected chlorogenate-related compounds (CGA) and all nicotianoside related compounds (Nicotianoside) detected were assembled in three single separated groups represented as three single nodes.

In addition, in order to look for putative links between the global gene expression modulators miRNAs, and metabolic pathways networks following infection the same correlation analyses were calculated including metabolites and miRNAs profiles during virus infection. Correlations of miRNAs and metabolites were calculated in the same way as described above setting the threshold at R>0.5 and *p* = 0.01 due to the smaller sample size and only metabolite-miRNA and miRNA-miRNA correlations are shown. Once again, correlation network was obtained from the infected plants which displayed 49 significant connections (Figure 8C) whilst 37 significant connections were observed in mock-inoculated samples (Figure 8D). Furthermore, there were 31 new correlations in infected samples not present in the mock-inoculated samples and in contrast 17 that were present in the mock-inoculated samples were lost after infection. Interestingly miR408 was the miRNA showing a hub behavior with the highest number of links to metabolites. In inoculated samples miR408 correlated with metabolites of the respiratory pathway citrate, isocitrate, glutarate and pyruvate. In contrast, these links were not evidenced in mock-inoculated samples.

## Discussion

Plant viral infections often produce a variety of disease symptoms, presumably by interfering with developmental processes, including altering cell division and/or expansion and symmetry, and/or by causing loss of pigmentation [50]. Many different approaches have been used to study plant-virus interactions including microarray, small RNA deep sequencing, infection of mutant plants, mutant viruses, virus replication/movement and NMR-based metabolomics [2], [39], [51], [52], [53], [54], [55].

Here, we obtained GC/LC-MS based metabolic profiles and miRNAs profiles during a compatible virus infection and both sets of data were analyzed in combination. We found that TMV infection triggers a systemic biphasic temporal regulation of metabolism. The first phase occurs 24 hours post-inoculation and is characterised by an increase in the levels of metabolites occurring in the absence of detectable virus in sampled tissue and potentially “priming the plant”. The second phase was characterized by both, up- and down-regulated metabolites and the number of the changes correlated with the increment in virus accumulation over time.

It seems reasonable to speculate that some metabolic changes observed at the early stage are probably the sensors and later propagators of the systemic signal that may orchestrate the plant defence after pathogen attack. Within the early stage group, there are at least four sets of molecules related with signalling that were altered by the infection. The hormone precursors benzoic acid and fatty acids (SA and JA respectively) were induced at 1 dpi, and it has been well demonstrated hormones changes after virus infection triggering stress responses [8], [11], [12], [56], [57]. GABA, a signalling molecule in mammals, was strongly up regulated at this phase and it has been postulated to play a similar role in plants [58]. However, it is important to note that in plants it also plays a highly important role in energy metabolism [59]. The fourth set of compounds were those of the ascorbate biosynthesis pathway implying an altered redox state, a change that has also previously been indicated to play an important role in the pathogen response [60], [61]. In agreement, altered levels of ascorbate derivatives (transient increase) and fatty acids were previously shown following TMV infection on tobacco plants on inoculated and systemic leaves by using an NMR based approach [52].

Following the initial phase (one dpi), the subsequent time point (five dpi) displayed the smallest number of statistical changes and eight, 15 and 22 dpi time points displayed increasing number of metabolic alterations in correlation with the level of infection. This result suggests that changes in late stages of infection depend upon the accumulation of virus and most likely are the consequence of the elevated level of resources required for the production of viral proteins [3], [42]. In full agreement, the observed negative correlation between CP levels and the levels of free amino acids plus the correlation between this results and the frequency of each amino acid in the CP composition (Figure 4A) strongly supports the hypothesis that virus accumulation modulates the metabolome to obtain resources.

Our observations of the miRNAs behavior (Figures 5 and 6) lead us to propose that two possibly different mechanisms could be acting at the early and late stage of infection to alter miRNAs accumulation, representing five dpi and 15–22 dpi respectively on the microarray data. In agreement to this hypothesis Hu et al [51] proposed recently the existence of at least more than one mechanism to explain the sRNAs alteration mediated by virus. The early stage (no virus presence) shows a cluster of miRNAs with down-accumulation and a concomitant increment in the mRNA level of one target gene tested, and the second, later stage of infection, includes higher levels of both miRNAs and miRNA-targets, as previously reported [22], [51], [62], [63], as well as viral proteins accumulation, suggesting that viral proteins inhibit miRNA-activity, for example, by sequestering sRNAs through the PTGS suppression activity of the replicase [37], [38], [39].

The early stage may include a transcriptional component, as implied by the coordinated reduction of the pre-miR166 and mature miR166 levels (Figure 7) also previously demonstrated by our group for the miR164a precursor on *Arabidopsis thaliana* [21].

The correlation analyses revealed a dense correlation network (metabolites and miRNAs) (Figure 8AC) in infected plants compared to the mock-inoculated ones (Figure 8BD). This information may indicate that the virus infection invokes the operation of, or increased flux through, a set of metabolic pathways that are highly interconnected. Consequently, it can be postulated that the infection modifies metabolite levels by means of key players which coordinate, or modulate, the levels of these components, for example miRNAs. Moreover, a few correlation modules were constant irrespective of the degree of infection. For example a secondary metabolism module, represented by nicotianoside and chlorogenic acid derivatives and flavonoids (Quercetin, Rutin and Kaempferol), was maintained across the experimental conditions described here (Figure 2A and 8AB), suggesting that the levels of these metabolites are either unaffected by viral infection or are modified in concert. However, comparison of the relative levels of the metabolites in question reveals that the first hypothesis is in fact correct. This is in close agreement with a previous proposal that these defense compounds are part of the autonomous defense mechanism [43].

Correlation analysis among miRNAs and metabolites revealed similar results. In particular, two correlation groups were observed in infected and mock-inoculated plants (Figure 8 CD). One constituted only by miRNAs and the second one involving both miRNA and metabolites. Interestingly, miR168, which regulates Ago1 (involved in the miRNA function process) [64], [65], in the mock-inoculated samples displayed several connections with the rest of the miRNA core. However, in the infected plant, miR168 only correlated with miRs 156/7 and 390, most probably as a consequence of the viral infection altering specific miRNA levels/activity. MiRNAs 397, 398 and 408 respond to nutrient deprivation, most particularly under copper and phosphate limitation [66]. miR408 (that together with miR403 are the only two down-accumulated miRNAs at late stage) can be considered a hub of our network analysis and, in infected leaves, it is linked to the TCA cycle intermediates citrate, isocitrate, glutarate and pyruvate. This latter association may imply a connection to the cellular respiration process through one of the miR408 targets (a plantacyanin-like protein (AT2G02850) that belongs to the phytocyanin family of secreted blue copper proteins) and/or reflex the copper homeostasis alteration on the miR398 regulating their target gene the COX5b protein, which is part of the respiratory chain complex III [47], [67]. Further experiments are required to establish this connection.

The basal defenses and the systemic signaling of viral associated molecular patterns (VAMPs) triggering innate immunity may be playing a role in the initiation of this early phase of miRNA alteration. In turn, this early phase may be orchestrating the antiviral defense in agreement to the suggestion of Ruiz-Ferrer and Voinnet [27]. Moreover, there are previous studies showing links between miRNA and hormones crostalk [12], [21], [56], [68] therefore allowing us to propose that changes in the mentioned metabolites (JA and SA precursors) that are infection-specific hubs on the response network (Figure 8 and 2A) may directly or indirectly trigger the alteration in systemic miRNA accumulation observed in the absence of virus (Figure 5, 5 dpi). In agreement with the proposal that the early phase should be mostly transcriptional, previous work of our and other laboratories detected elements responsive to SA and/or JA in several promoters of the miRNAs that compose the cluster altered at the beginning of the infection stage [21], [46]. It is important to mention that the miRNAs that form cluster A are part of a miRNAs group described as stress regulated miRNAs, responsive to several different kind of stresses [46], [47], [48], [69], [70]. The fact that ShMV (mild virus) produces the early miRNA alteration at a delayed stage in some of the five tested miRNAs in comparison to TMV (severe virus) (Figure 6) and the fact that ShMV produces light symptoms may indicate that this early alteration is important to either the severity itself or to the timing required for symptom development.

In conclusion we have showed a global and more importantly bi-phased series of changes in metabolites and several miRNAs produced following TMV infection, in systemic leaves. Several of the metabolites increased at this first stage were related to hormone production and some of them could be proposed to propagate the phloem signal within the systemic leaf cells. In the case of miRNA the early phase, without virus accumulation, may respond to a systemic signal that probably acts transcriptionally on miRNA genes to produce the mature miRNAs alteration. However, further experiments are required in order to uncover which metabolites/miRNAs play causal biological roles during the virus infection. As a consequence, further knowledge of the role of miRNA in regulating metabolic pathways and/or *vice versa* will likely be a great aid in understanding the role of miRNAs during host-pathogen interactions.

## Materials and Methods

### Plants growing conditions, viral infections and quantification

All *N. tabacum* plants (Xhanti nn) were grown in a greenhouse with temperatures ranging from 20 to 26°C. A single expanded leaf of each plant (leaf 4 to 6 stage) was dusted with carborundum, 20 µl of semi-purified TMV virus in 20 mM NaHPO_4_ (pH 7) was added, and the surface of the leaf was gently abraded. Two level of inoculums were used, the experiment mentioned in figure 1, 2, 3, 4, 5 and 8 used more than 200 Local Lesion (LL) inoculums, the experiment of figure 6 and 7 used approximately 20 LL inoculums for both viruses TMV and ShMV. The third leaf above the inoculated one was collected (leaf number eight) (Figure 1). For quantification of viral proteins, total proteins were extracted and quantified using Quick Start Bradford Protein Assay (Bio-Rad). For viral detection ELISA experiments were performed as described previously [71].

### Metabolic analysis

Metabolite extractions were done as previously described [72]. For GC-MS, derivatization and GC-time of flight-MS (GC-TOF-MS) analyses were carried out as described previously [73]. The GC-TOF-MS system was composed of a CTC CombiPAL autosampler, an Agilent 6890N GC and a LECO Pegasus III time-of-flight mass spectrometer running in EI^+^mode. Metabolites were identified by comparison to database entries of authentic standards and relative quantification was performed as described in [74].

For secondary metabolites extraction for the LC-MS injection were done as follows: 50 mg of frozen plant tissues were homogenized in 10 µL of extraction solvent (80% methanol with 10 µg/ml of isovitexin, EXTRASYNTHASE, Genay Cedex, France) per mg of fresh weight of tissue in a mixer mill MM300 (Retsch, Haan, Germany) for 3 min at 30 Hz. After centrifugation, 400 µl were transferred to new tube for evaporation. Dried pellets were resolved by 400 µl of 80% Methanol. After filtration by centrifugal device NanoSep MF GHP 45 µm filter (PALL, NY, USA) with centrifugation at 3,000 g. The filtrates were immediately used for injection. HPLC-MS analysis was performed on HPLC system Surveyor (Thermo Finnigan, USA) coupled to Finnigan LCQ-Deca system (Thermo Finnigan, USA). Five µl of the plant extract was injected onto the column (Luna 3 µ C18(2), 100A, Phenomenex, Torrance, CA, USA) with elution buffer A (water with 0.1% of formic acid) and buffer B (acetonitrile with 0.1% formic acid). Flow rate was 0.2 ml/min. Separation conditions were as follows: 0–2 min, isocratic elution with 100% A; 2–4 min, linear gradient from 0% to 15% B; 4–14 min, linear gradient from 15% to 32% B; 14–19 min, linear gradient from 32% to 50% B; 19–21 min, isocratic elution with 100% B. The mass analyzer was used for the detection in a negative/positive ion scanning mode with the following setting: capillary temperature, 350°C; source voltage, 5.00 kV and capillary voltage, 60.0 V in positive ion detection and −10.0 V in negative ion detection. Peak areas were integrated using Xcalibur software 2.1 (Thermo Finnigan, USA). Rutin and chlorogenic acid were used for the identification of the peaks in the plant extracts based on co-elution profile for retention times and mass fragmentation by tandem MS analysis. Other peaks which are not available of standard compounds were annotated by comparing their *m/z* values and MS^2^ fragmentation patterns with reference compounds, the reported data [75], [76], [77] and metabolite databases [78], [79].

Tagfinder software for GC-MS results and Xcalibur software for LC-MS was used to assign the peaks identity [80]. Metabolites abundance was calculated based in the Ribitol or Isoxitexin intensity and tissue weight. Averages, standard errors and Student's t-tests calculated. VANTED software was used to show and analyze metabolites abundance and statistical changes in a network, manually draw based on published databases and papers [81].

### Correlation analysis

Non-parametric Spearman correlation analyses were calculated using Infostat software (InfoStat software [InfoStat version 2008. Grupo InfoStat, FCA, Universidad Nacional de Córdoba, Argentina]), filtered at *p* = 0.001 for metabolites and *p* = 0.01 for miRNA data. Significant correlations were drawn using netdraw (http://www.analytictech.com/).

### RNA extraction and quantitative real-time polymerase chain reaction

For relative quantification of miRNAs and target mRNAs accumulation levels, experiments were carried out using five biological replicates. The RT-qPCR performed complies with the MIQE requirements, for the detailed data on each experiment see Supplemental Figure S7. Total RNA was isolated from leaves using TRIZOL® Reagent (Invitrogen), quantified using spectrophotometer (NanoDropTechnologies), and treated with DNase I (Invitrogen). First-strand cDNA was synthesized either using Superscript III (Invitrogen) and stem-loop specific primers (for miRNAs accumulation analysis) or MMLVI and oligo d(T)20 primers (for targets mRNA accumulation analysis). Real-time qPCR to detect miRNAs was performed as described by Chen et al [82]. The oligonucleotide primer sets for mRNA quantification were designed using Primer Express 2.0 software (Applied Biosystems) to amplify a fragment containing the miRNA target recognition site. All qPCR reactions were performed using *Platinum* **®** *Taq* DNA Polymerase (Invitrogen) and *SYBR* **®**Green stain (Roche). qPCR data analysis and primer efficiencies were obtained using LinRegPCR Software [83]. To select the reference genes a stability test was performed using GeNorm, NormFinder and Bestkeeper algorithms on three candidate genes (Supplemental Figure S8). The candidates genes were Tabacco elongation factor-1α (EF-1α, SGN-U446573), tobacco Actin gene (SGN-U431117) and Ubiquitin 3 gene (Ubi-3, GB: X58253). Based on the result observed EF-1α was selected and used as internal control given that it was the most consistent along the three algorithms used and it was in agreement to the reference genes proposed by Lilly et at [84] for virus infections even thought the three selected genes show similar stability. Relative expression ratios and statistical analysis were performed by using FgStatistics interface (Di Rienzo J.A, (2009). Statistical software for the analysis of experiments of functional genomics. http://sites.google.com/site/fgStatistics/). This software makes use of Pfaffl algorithm [85] to calculate the expression ratio of a given gene in a improved multivariate user friendly interface and calculate the statistic significance using a permutation test (See Supplemental Figure S7). The cut-off for statistically significant differences was set as * means p-value<0.1; ** means p-value<0.05 and *** means p-value<0.01. All primer sets are listed in Supplemental Figure S9 [85].

### sRNAs extraction and MicroArray Assay

High-quality sRNAs were extracted from tobacco leaves using miRNAVana kit (Ambion, USA) following manufacturer's indications and then quantified using a spectrophotometer (NanoDropTechnologies). Combimatrix CustomArray™ 4X2K miRNA array was used, each slide has four identical array sectors that can be hybridized with different microRNA samples simultaneously, each array sector carry anti-sense oligonucleotide probes with a median length of 22 nucleotides and an average Tm of 55 to 60°C. The 2000 probes on each array/sector contain the complete set of plants mature miRNAs available on MirBase 9.0 in triplicates along the array (*Arabidosis thaliana* miRNAs probes are repeated four times). The negative controls for RNA degradation and positive controls (U6 and tRNAs) were used to accept array data. Preparation and labeling of miRNA samples for hybridization were performed following manufacturer's protocols (Combimatrix). Images were collected and digitalized using a laser scanner (arrayWoRx^e^ Standard Biochip Reader; Applied Precision, WA, USA). The Microarray Imager software from Combimatrix was used in order to extract raw data from images. A total of n = 6 plants were separately tested for each time point (5, 15 and 22 dpi) of each treatment (mock- and virus-inoculation). Samples were randomly assigned in blocks from the same dpi (mock/virus inoculations) into the three chips used (each with four independent chambers or arrays), which were subsequently stripped and reused twice following manufacturer's protocol. Therefore a total of 36 arrays, each one belonging to one biological replicate were performed. Raw fluorescence values were normalized (As suggested by Combimatrix), background-substracted and a mean value from technical replicates for each miRNA sub-type for each species-specific probe (e.g., ath|miR156a) for every array was calculated. Afterwards a median value was obtained for every single miRNA type and species-specific probe (e.g., ath|miR156) at 5, 15 and 22 days post-inoculation (dpi) for all arrays and each treatment. Median values were only taken into account in the case of the existence of at least three independent arrays showing mean values above the background for each miRNA and probe. Later a log-2 quotient between infected and mock-inoculated was calculated for each miRNA type and species-specific probe fluorescence value. A median value was then calculated in order to obtain a single value for each miRNA for all probes spotted in the arrays. MiRNAs belonging to closely related families with similar mature sequences were put together for the calculations. The results were visualized as a log-2 false-color scale (MultiExperiment Viewer Version 4.2 August 1st, 2008) [86].

## Supporting Information

Figure S1 **Virus severity comparison.** Systemic symptoms on infected tobacco plants at 30 days post infection (TMV, and ShMV respectively). Percentage of plants showing systemic symptoms observed on inoculated plants with the selected viruses in two independent experiments at different days post infection, with a minimum of ten plants per assay. Similar virus concentrations were used (∼2.5 mg/ml) as inocula. Number of plants with flowers and the average height of plants quantified 30 days post infection in two independent infection assays.
(XLS)

Figure S2 **Excel table showing the data used to build** **Figure 2** **and** **3** **.** **A**) Metabolite changes detected in systemic leaves from TMV and Mock-inoculated plants, data is presented normalized for each time point and as a ratio between the levels of each metabolite in infected versus the mock-inoculated plants in each time point, this ratios indicate the metabolic alteration produced by the virus infection at each time point. **B**) Metabolite changes detected in Mock-Inoculated plants along the time course of the experiment, data is showed as the ratio between data of each time point of mock-inoculated samples versus the first dpi of mock-inoculated. This ratio indicates the metabolic changes produced as a consequence of the sampled leaf development along the time of the experiment. In addition in both A and B tables information about the peak assignment is display.
(XLS)

Figure S3 **CP-TMV detection by RT-PCR analysis in 5 and 22 dpi TMV and mock infected tobacco plants.** It is observed that at 5 dpi there is no detectable virus in sampled tissue. For RT-PCR, first-strand cDNA was synthesized using M-MLV RT (Promega) and random primers according to manufacturer's instructions (Promega). The oligonucleotide primer set used for CP detection were designed using Vector NTI Software. The PCR was performed using Taq (Invitrogen) under the following program: 1× 5 min 95°C, 30× 15 sec 95°C, 30 sec 58°C, 30 sec 72°C. The product were analized in 1.2% Agarose gel.
(TIF)

Figure S4 **Excel table showing the data used to build** **Figure 5** **.** **A**) Fluorescence values extracted from microarray hybridizations using sRNA obtained fractions from systemic leaves of infected (TMV) or buffer-inoculated (MOCK) *N. tabacum* plants. Raw fluorescence values were normalized, background-substracted and a mean value from technical replicates for each miRNA sub-type for each species-specific probe (e.g., ath|miR156a) for every array was calculated. Afterwards a median value was obtained for every single miRNA type and species-specific probe (e.g., ath|miR156) at 5, 15 and 22 days post-inoculation (dpi) for all arrays and each treatment. Median values were only taken into account in the case of the existence of at least three independent arrays showing mean values above the background for each miRNA and probe. The “no data” annotation indicates lack of sufficient mean values. **B**) Using the data presented in A a median value was then calculated in order to obtain a single value for each miRNA for all probes spotted in the arrays. MiRNAs belonging to closely related families with similar mature sequences were put together for the calculations. There after a log-2 quotient between infected and mock-inoculated was calculated for each miRNA.
(TIF)

Figure S5 **TMV-CP cuantitative detection by RT-qPCR relative quantification analysis at 6, 11 and 22 dpi TMV and mock infected tobacco plants.** Ratios were obtained between mock and TMV infected plants from each day post infection. It is observed that at 11 dpi low but significative amounts of CP is detected and at 22 dpi a severe infection shows high amounts of TMV CP. *** indicates p-value<0.001.
(TIF)

Figure S6 **miRNA Targets genes validation.** (**A–B**) Alignments of the miRNA-target sites between tobacco genes: SGN-U435399, SGN-U439906, and SGN-U428805 mRNAs and reported *Arabidopsis* miRNA-target genes. (**C**) miR156, miR166 and miR171 cleavage sites in target genes SGN-U435399, SGN-U439906, and SGN-U428805 mRNAs, respectively, determined by a modified RNA ligase-mediated RACE. The frequency of RACE clones corresponding to each cleavage site (arrows) is shown with the number of clones matching the target message. Aligned are the reported Arabidopsis target sites. **Methods:** To map the internal cleavage site in putative targets of tobacco mRNA, RNA ligase-mediated rapid amplification of cDNA ends (RLM-RACE) was done using the GeneRacer Kit (Invitrogen, Carlsbad, CA). A modified procedure for RLM-RACE was carried out as described previously by Llave et al. (Llave C, Xie Z, Kasschau KD, Carrington JC (2002) Science 297:2053–20562002). Total RNA was isolated from 4-week old plants and Poly(A)+ mRNA was purified using an Oligotex mRNA Midi Kit (Qiagen, Germany). Two nested PCRs were done to amplify the DNA fragment, which was subsequently cloned into pGEM-T Easy vector (Promega, Madison, WI) for sequencing.
(TIF)

Figure S7 **Experimental conditions used in Quantitative Real Time PCR Experiments based on MIQE requirements.**
(TIF)

Figure S8 **Stability analysis of candidate reference genes in 6 and 11 dpi, TMV, SHMV and mock infected tobacco leaves.** The housekeeping genes were ranked according to their expression stability by (A) geNorm, (B) Normfinder and (C) BestKeeper statistical tools. In the three plots, genes were ordered from least (left) to most (right) stable. (C, bottom) Pair-wise correlation analysis between the candidate reference genes and the calculated BestKeeper index were highlighted in grey. (D) Ct values of the three houskeeping genes EF-1α, Ubi-3 and Actin, obtained by RT-qPCR and LinReg data analysis. Ct values resulting from RT-qPCR and LinReg software were used for expression stability analysis using the Microsoft Excel based tools geNorm 3.5 (Vandesompele et al 2002), Normfinder 0.953 (Andersen et al 2004) and Bestkeeper v1 (Pfaffl et al, 2004) according to the developer's instructions. Vandesompele J, De Preter K, Pattyn F, Poppe B, Van Roy N, De Paepe A, Speleman F: Accurate normalization of real-time quantitative RT-PCR data by geometric averaging of multiple internal control genes. Genome Biol 2002, 3(7):RESEARCH0034 Andersen CL, Jensen JL, Orntoft TF: Normalization of real-time quantitative reverse transcription-PCR data: a model-based variance estimation approach to identify genes suited for normalization, applied to bladder and colon cancer data sets. Cancer Res 2004, 64:5245–5250. Pfaffl MW, Tichopad A, Prgomet C, Neuvians TP: Determination of stable housekeeping genes, differentially regulated target genes and sample integrity: BestKeeper–Excel-based tool using pair-wise correlations. Biotechnol Lett 2004, 26:509–515.
(DOC)

Figure S9 **List of used primers.**
(DOC)
