# Supplementary figures and images for: Metabolic and miRNA Profiling of TMV Infected Plants Reveals Biphasic Temporal Changes
Source: PLoS One. 2011 Dec 12;6(12):e28466. doi: 10.1371/journal.pone.0028466 (PMC3236191; doi:10.1371/journal.pone.0028466)

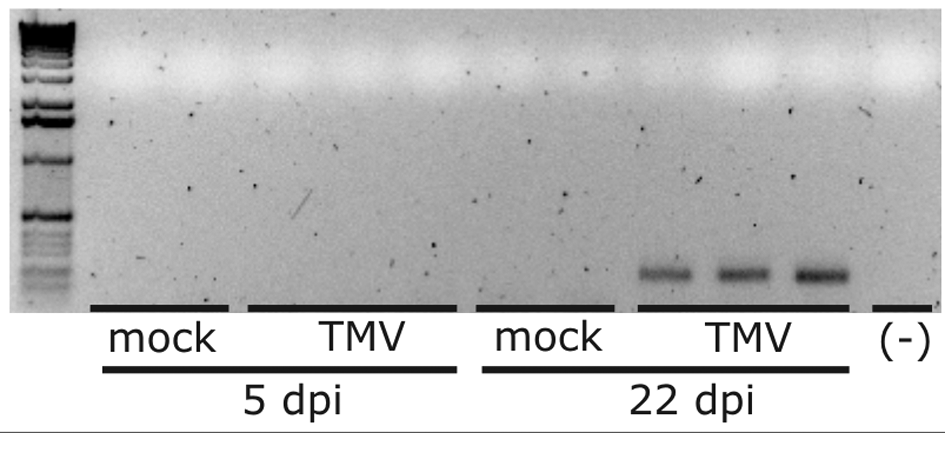

Supplement: Figure S3 — CP-TMV detection by RT-PCR analysis in 5 and 22 dpi TMV and mock infected tobacco plants. It is observed that at 5 dpi there is no detectable virus in sampled tissue. For RT-PCR, first-strand cDNA was synthesized using M-MLV RT (Promega) and random primers according to manufacturer's instructions (Promega). The oligonucleotide primer set used for CP detection were designed using Vector NTI Software. The PCR was performed using Taq (Invitrogen) under the following program: 1× 5 min 95°C, 30× 15 sec 95°C, 30 sec 58°C, 30 sec 72°C. The product were analized in 1.2% Agarose gel. (TIF) [file pone.0028466.s003.tif]

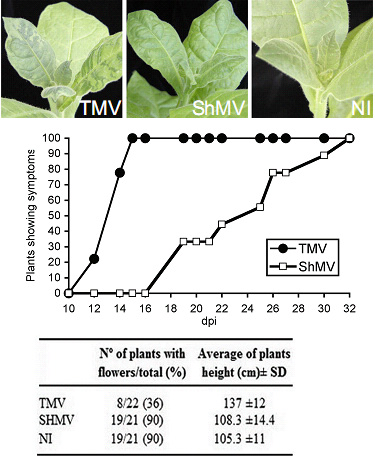

Supplement: Figure S4 — Excel table showing the data used to build Figure 5 . A) Fluorescence values extracted from microarray hybridizations using sRNA obtained fractions from systemic leaves of infected (TMV) or buffer-inoculated (MOCK) N. tabacum plants. Raw fluorescence values were normalized, background-substracted and a mean value from technical replicates for each miRNA sub-type for each species-specific probe (e.g., ath|miR156a) for every array was calculated. Afterwards a median value was obtained for every single miRNA type and species-specific probe (e.g., ath|miR156) at 5, 15 and 22 days post-inoculation (dpi) for all arrays and each treatment. Median values were only taken into account in the case of the existence of at least three independent arrays showing mean values above the background for each miRNA and probe. The “no data” annotation indicates lack of sufficient mean values. B) Using the data presented in A a median value was then calculated in order to obtain a single value for each miRNA for all probes spotted in the arrays. MiRNAs belonging to closely related families with similar mature sequences were put together for the calculations. There after a log-2 quotient between infected and mock-inoculated was calculated for each miRNA. (TIF) [file pone.0028466.s004.tif]

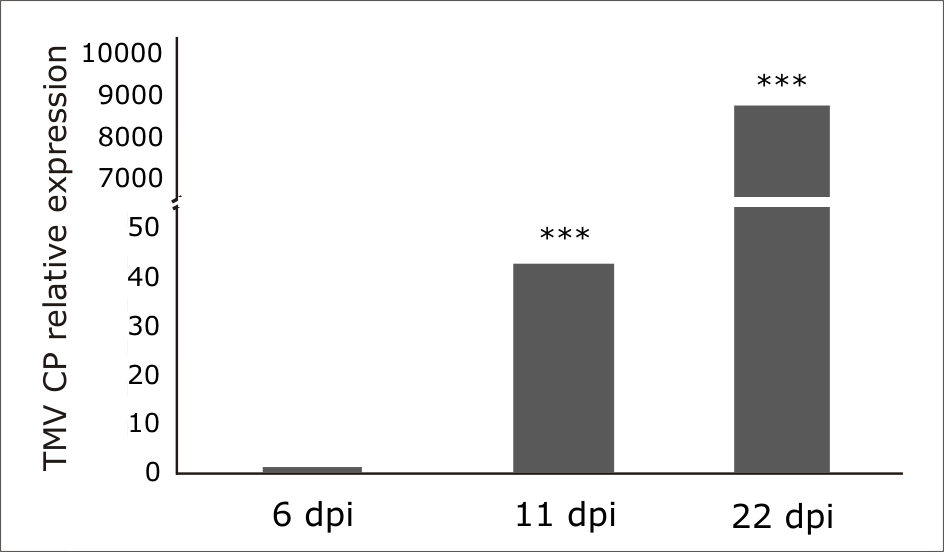

Supplement: Figure S5 — TMV-CP cuantitative detection by RT-qPCR relative quantification analysis at 6, 11 and 22 dpi TMV and mock infected tobacco plants. Ratios were obtained between mock and TMV infected plants from each day post infection. It is observed that at 11 dpi low but significative amounts of CP is detected and at 22 dpi a severe infection shows high amounts of TMV CP. *** indicates p-value<0.001. (TIF) [file pone.0028466.s005.tif]

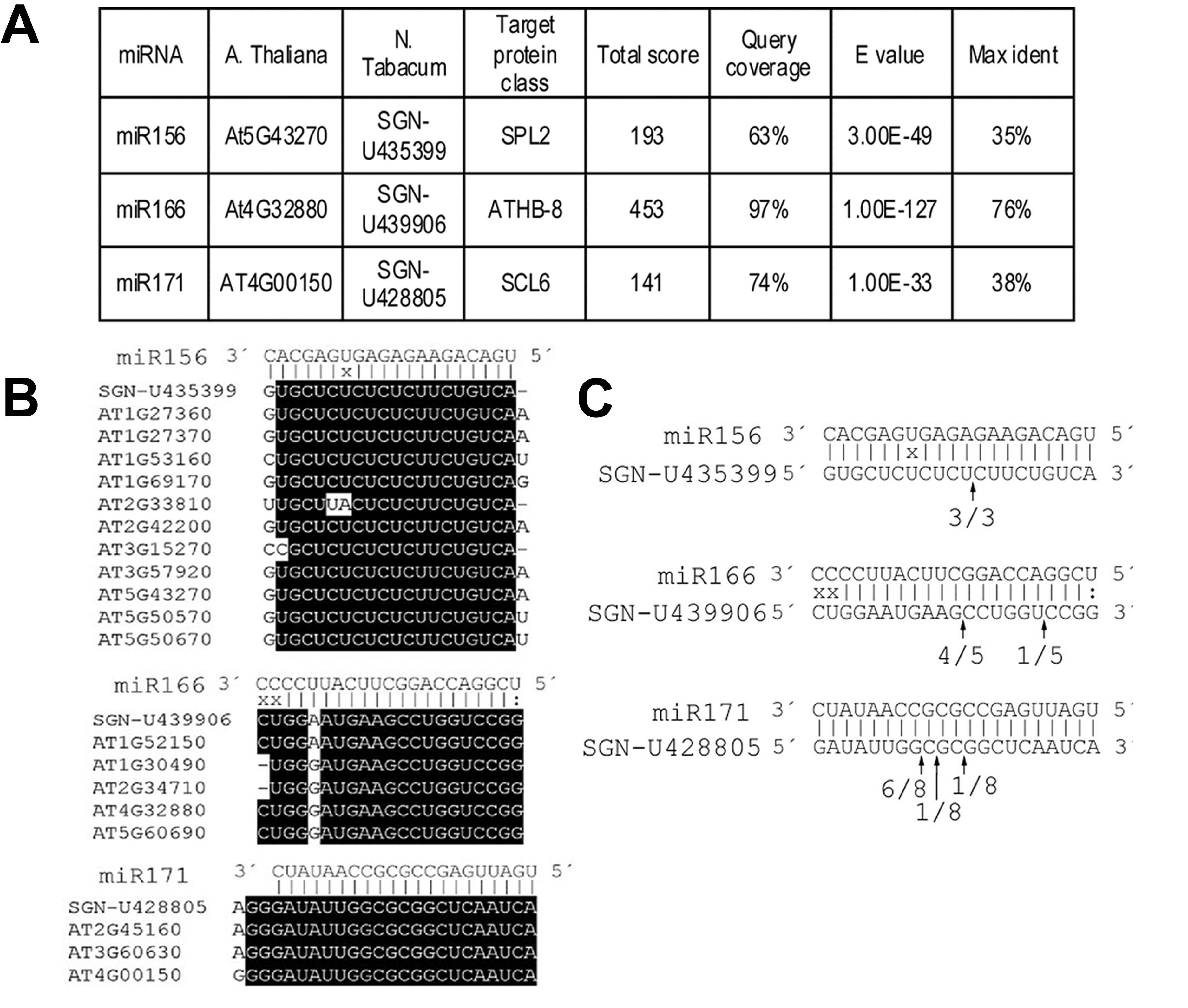

Supplement: Figure S6 — miRNA Targets genes validation. (A–B) Alignments of the miRNA-target sites between tobacco genes: SGN-U435399, SGN-U439906, and SGN-U428805 mRNAs and reported Arabidopsis miRNA-target genes. (C) miR156, miR166 and miR171 cleavage sites in target genes SGN-U435399, SGN-U439906, and SGN-U428805 mRNAs, respectively, determined by a modified RNA ligase-mediated RACE. The frequency of RACE clones corresponding to each cleavage site (arrows) is shown with the number of clones matching the target message. Aligned are the reported Arabidopsis target sites. Methods: To map the internal cleavage site in putative targets of tobacco mRNA, RNA ligase-mediated rapid amplification of cDNA ends (RLM-RACE) was done using the GeneRacer Kit (Invitrogen, Carlsbad, CA). A modified procedure for RLM-RACE was carried out as described previously by Llave et al. (Llave C, Xie Z, Kasschau KD, Carrington JC (2002) Science 297:2053–20562002). Total RNA was isolated from 4-week old plants and Poly(A)+ mRNA was purified using an Oligotex mRNA Midi Kit (Qiagen, Germany). Two nested PCRs were done to amplify the DNA fragment, which was subsequently cloned into pGEM-T Easy vector (Promega, Madison, WI) for sequencing. (TIF) [file pone.0028466.s006.tif]

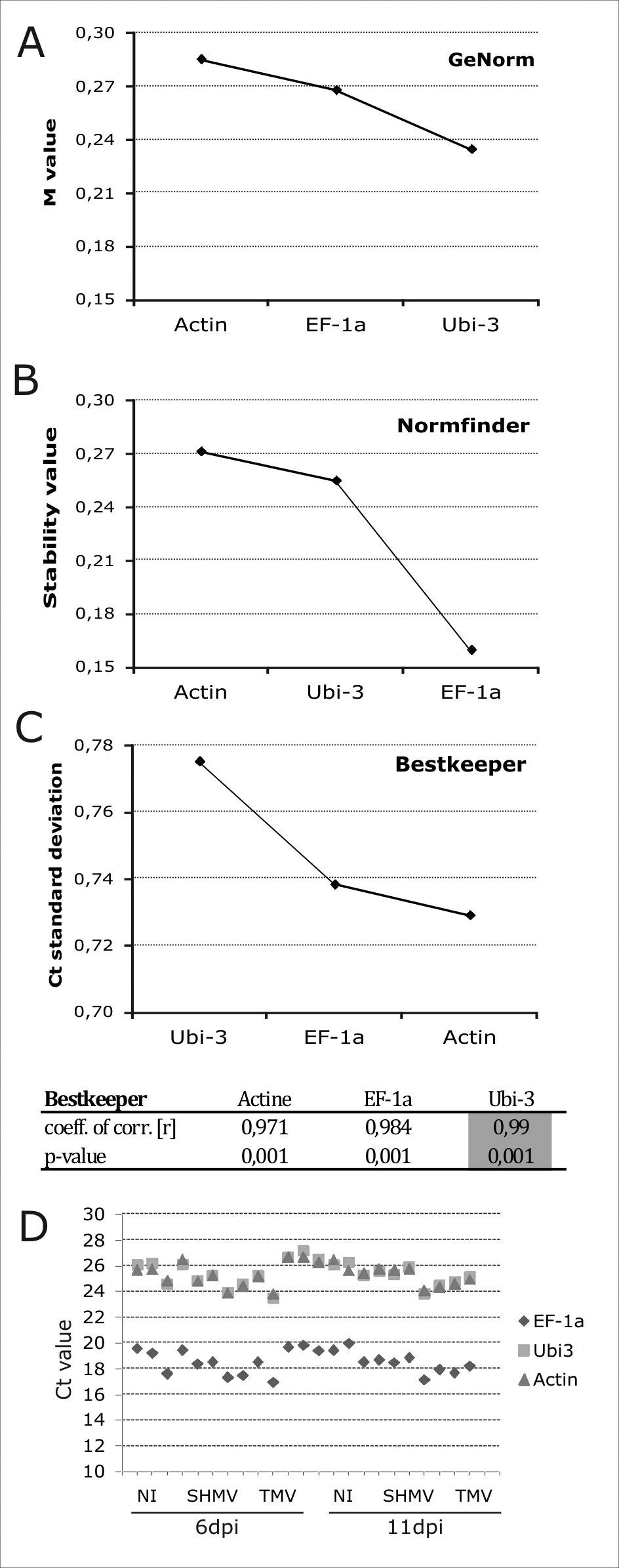

Supplement: Figure S7 — Experimental conditions used in Quantitative Real Time PCR Experiments based on MIQE requirements. (TIF) [file pone.0028466.s007.tif]
