# Supplementary material for: Metabolic and miRNA Profiling of TMV Infected Plants Reveals Biphasic Temporal Changes
Source: PLoS One. 2011 Dec 12;6(12):e28466. doi: 10.1371/journal.pone.0028466 (PMC3236191; doi:10.1371/journal.pone.0028466)
Supplement: Figure S8 — Stability analysis of candidate reference genes in 6 and 11 dpi, TMV, SHMV and mock infected tobacco leaves. The housekeeping genes were ranked according to their expression stability by (A) geNorm, (B) Normfinder and (C) BestKeeper statistical tools. In the three plots, genes were ordered from least (left) to most (right) stable. (C, bottom) Pair-wise correlation analysis between the candidate reference genes and the calculated BestKeeper index were highlighted in grey. (D) Ct values of the three houskeeping genes EF-1α, Ubi-3 and Actin, obtained by RT-qPCR and LinReg data analysis. Ct values resulting from RT-qPCR and LinReg software were used for expression stability analysis using the Microsoft Excel based tools geNorm 3.5 (Vandesompele et al 2002), Normfinder 0.953 (Andersen et al 2004) and Bestkeeper v1 (Pfaffl et al, 2004) according to the developer's instructions. Vandesompele J, De Preter K, Pattyn F, Poppe B, Van Roy N, De Paepe A, Speleman F: Accurate normalization of real-time quantitative RT-PCR data by geometric averaging of multiple internal control genes. Genome Biol 2002, 3(7):RESEARCH0034 Andersen CL, Jensen JL, Orntoft TF: Normalization of real-time quantitative reverse transcription-PCR data: a model-based variance estimation approach to identify genes suited for normalization, applied to bladder and colon cancer data sets. Cancer Res 2004, 64:5245–5250. Pfaffl MW, Tichopad A, Prgomet C, Neuvians TP: Determination of stable housekeeping genes, differentially regulated target genes and sample integrity: BestKeeper–Excel-based tool using pair-wise correlations. Biotechnol Lett 2004, 26:509–515. (DOC) [file pone.0028466.s008.doc]

**Experimental conditions used in Quantitative Real Time PCR Experiments**

**A: RT-qPCR to detect mature miRNAs.**

| **Experimental design** |  |
| --- | --- |
| Control groups | Mock infected plants (2 groups 6 and 11 dpi) |
| Treatment groups | TMV and ShMV infected plants 6 and 11 dpi (four groups) |
|  |  |
| **Sample** |  |
| Type of sample | Tobacco leaves |
| Processing procedure | Liquid nitrogen homogenization |
| Sample frozen conditions | -80ºC |
|  |  |
| **RNA extraction** |  |
| Procedure | Acid Phenol extraction |
| Reagents | TRIzol(Invitrogen) |
| Details of Dnasa treatment | DNAsa I Amp Grade, 15 min at room temperature |
| Contamination assesment | <3% |
| Nucleic acid quantification | Absorbance at 260nm |
| Instrument and method | NanoDrop instrument |
| Purity( A260/ A 280) | >1.8 |
| RNA integrity | Analyzed by agarose gel electrophoresis |
|  |  |
| **Reverse transcription** |  |
| Complete reaction conditions | Protocol modified from Chen et al, 2005. |
| Amount of RNA and reaction volume | 100ng of RNA, 20 µl |
| Priming oligonucleotide | stem-loop specific primers for each miRNAs and Specific primer for EF1α reference gene. |
| Reverse transcriptase | SuperScript® III Reverse Transcriptase, 60 U per reaction |
| Temp and time | 30 min 16ºC , (30 seg 30ºC, 30 seg 42ºC, 30 seg 50ºC x 60 cycles), 5 min 85ºC |
|  |  |
| **qPCR protocol** |  |
| Complete reaction conditions | 5 min 95 ºC , (30 seg 95ºC, 1 min 60 ºC) x 40 cycles |
| Reaction volume and amount of cDNA | 20µl reaction, 20-200 ng de RNA |
| Primer, Mg and dNTPs concentration | 3mM Mg2+, 200nM primers, 0,2 mM de dNTPs |
| Polymerase | Taq platinum, Invitrogen |
| Buffer | 20 mM Tris-HCL ( ph 8.4), 50 mM Kcl |
| Manufacturer of qPCR instrument | ABI 7500, Applied Biosystems |
|  |  |
| **qPCR validation** |  |
| Specificity | Analysed by agarose gel and Melting Curve parameters on each qPCR run. |
| Method of PCR efficiency calculation | Mean PCR efficiency per amplicon calculated by LingRegPCR program (Ramakers et al, 2003). |
|  |  |
| **Data analysis** |  |
| qPCR analysis program | LinRegPCR program |
| Method of Cq determination | LinRegPCR program |
| Outlier identification | LinRegPCR program |
| Justification of number and choice of reference genes | 3 references genes were tested (Actin, Ubi-3 and EF1α) for stability using three stability determination algorithms. EF1 α gene was selected as reference gene. |
| Description of normalization methods | Pfaffl M.W et al, 2002.  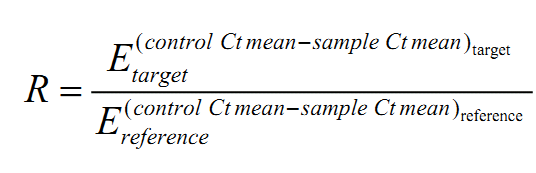  Implemented in a multivariate user-friendly interface by FGstatistic Software. |
| Number of technical replicates | 3 |
| Statistical method | Permutation test |
| Software | Fg Satistics |
| Repeatability (intra-assay variation) C_q_ SD error | 0.092 |

**B: RT-qPCR to detect mRNAs (Target Genes)**

| **Experimental design** |  |
| --- | --- |
| Control groups | Mock infected plants (2 groups 6 and 11 dpi) |
| Treatment groups | TMV and ShMV infected plants 6 and 11 dpi ( four groups) |
|  |  |
| **Sample** |  |
| Type of sample | Tobacco leaves |
| Procesing procedure | Nitrogen liquid homogenization |
| Sample frozen conditions | -80ºC |
|  |  |
| **RNA extraction** |  |
| Procedure | Acid Phenol extraction |
| Reagents | TRIzol (Invitrogen) |
| Details of Dnasa treatment | DNAsa I Amp Grade, 15 min at room temperature |
| Contamination assesment | <3% |
| Nucleic acid quantification | Absorbance at 260nm |
| Instrument and method | NanoDrop instrument |
| Purity( A260/ A 280) | >1.8 |
| RNA integrity | Analyzed by agarose gel electrophoresis |
|  |  |
| **Reverse transcription** |  |
| Complete reaction conditions | Reaction was performed as described by the Invitrogen ® instructions. |
| Amount of RNA and reaction volume | 1µg of RNA, 20 µl |
| Priming oligonucleotide | oligo d(T)20 primers (Invitrogen®) . |
| Reverse transcriptase | MMLVI (Invitrogen). |
| Temp and time | 1 hour, 50 ºC |
|  |  |
| **qPCR protocol** |  |
| Complete reaction conditions | 5 min 95 ºC , (30 seg 95ºC, 1 min 60 ºC) x 40 cycles |
| Reaction volume and amount of cDNA | 20µl reaction, 20-200 ng de RNA |
| Primer, Mg and dNTPs concentration | 3mM Mg2+, 200nM primers, 0,2 mM de dNTPs |
| Polymerase | Taq platinum, Invitrogen |
| Buffer | 20 mM Tris-HCL ( ph 8.4), 50 mM Kcl |
| Manufacturer of qPCR instrument | ABI 7500, Applied Biosystems |
|  |  |
| **qPCR validation** |  |
| Specificity | Analysed by agarose gel and Melting Curve parameters on each qPCR run. |
| Method of PCR efficiency calculation | Mean PCR efficiency per amplicon calculated by LingRegPCR program (Ramakers et al, 2003). |
|  |  |
| **Data analysis** |  |
| qPCR analysis program | LinRegPCR program |
| Method of Cq determination | LinRegPCR program |
| Outlier identification | LinRegPCR program |
| Justification of number and choice of reference genes | 3 references genes tested ( Actin, Ubi-3 and EF1α) for stability using three stability algorithms. EF1 α gene was selected as reference gene. |
| Description of normalization methods | Pfaffl M.W et al, 2002  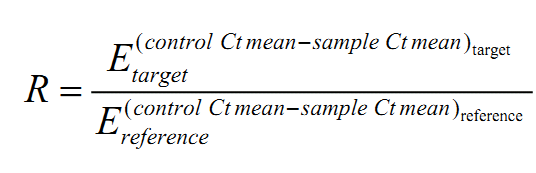  Implemented in a multivariate user-friendly interface by FGstatistic Software. |
| Number of technical replicates | 3 |
| Statistical method | Permutation test |
| Software | Fg Satistics |
| Repeatability (intra-assay variation) C_q_ SD error | 0.17 |

1) **Assumption-free analysis of quantitative real-time PCR data**

Ramakers C, Ruijter JM, Deprez RH, Moorman AF. (2003) Neurosci Lett  2003 Mar 13;339(1): 62-66

2**Relative expression software tool (REST©) for group-wise comparison and statistical analysis of**

**relative expression results in real-time PCR.**

Michael W. Pfaffl, GrahamW. Horgan and Leo Dempfle. Nucleic Acids Research, 2002, Vol. 30, No. 9 00

3) **The MIQE Guidelines:Minimum Information for Publication of Quantitative**

**Real-Time PCR Experiments** .

Stephen A. Bustin,Vladimir Benes,

Jeremy A. Garson, Jan Hellemans, Jim Huggett, Mikael Kubista, Reinhold Mueller, Tania Nolan, Michael W. Pfaffl, Gregory L. Shipley, Jo Vandesompele, 5and Carl T. Wittwer.

Clinical Chemistry 55:4 611–622 (2009)

4**) Real-time quantification of microRNAs by stem–loop RT–PCR**

Caifu Chen*, Dana A. Ridzon, Adam J. Broomer, Zhaohui Zhou, Danny H. Lee,

Julie T. Nguyen, Maura Barbisin, Nan Lan Xu, Vikram R. Mahuvakar, Mark R. Andersen,

Kai Qin Lao, Kenneth J. Livak and Karl J. Guegler

Applied Biosystems, 850 Lincoln Centre Drive, Foster City, CA 94404, USA
