# Supplementary material for: Metabolic and miRNA Profiling of TMV Infected Plants Reveals Biphasic Temporal Changes
Source: PLoS One. 2011 Dec 12;6(12):e28466. doi: 10.1371/journal.pone.0028466 (PMC3236191; doi:10.1371/journal.pone.0028466)
Supplement: Figure S9 — List of used primers. (DOC) [file pone.0028466.s009.doc]

List of used primers.

| **Primer name** | **Sequence** |
| --- | --- |
| **Target-156 Forward** | GCAGTCATTATTGCCAGGGATCC |
| **Target-156 Reverse** | TGAAACAGTCTCAGGCTCGGATG |
| **Target-166 Forward** | CCACTTGACATCTCAGCATCCGC |
| **Target-166 Reverse** | CCGTGCAACCATGAGAAATAGCA |
| **Target-171 Forward** | TACAACTACAGCAGCAGCAGCAGC |
| **Target-171 Reverse** | CTCCTTGCAATAAAAAGCAGCCC |
| **Pre-miR166a Forward** | GTTGAGGAGAATGTCGTCTGG |
| **Pre-miR166a Reverse** | GTTGAGGGGAATGAAGCCT |
| **156-F** | CGCGTGACAGAAGAGAGT |
| **164-F** | CTTCGTGCACGTGCCCTGCTT |
| **165-F** | TCGCGTCGGACCAGGCTTCAT |
| **167-F** | TCGCGTGAAGCTGCCAGCAT |
| **171-F** | TCGCGTGATTGAGCCGCGCC |
| **Universal** | GTGCAGGGTCCGAGGT |
| **RT-157** | GTCGTATCCAGTGCAGGGTCCGAGGTATTCGCACTGGATACGACGTGC |
| **RT-164** | GTCGTATCCAGTGCAGGGTCCGAGGTATTCGCACTGGATACGACTGCA |
| **RT-166** | GTCGTATCCAGTGCAGGGTCCGAGGTATTCGCACTGGATACGACGGGG |
| **RT-167** | GTCGTATCCAGTGCAGGGTCCGAGGTATTCGCACTGGATACGACTAGATC |
| **RT-171** | GTCGTATCCAGTGCAGGGTCCGAGGTATTCGCACTGGATACGACGATA |
| **TD1-chPrec: (SGNU439040) FWD** | CCGCCGGCTAAATCTCCACATC |
| **TD1-chPrec: (SGNU439040) REV** | CAATGGGGGAGCTTTTAGCCGT |
| **AAAtransf3 (SGNU444353) Fwd** | TGGTGTCCGTGGACGAATTG3 |
| **AAAtransf3 *(SGNU444353) REV*** | CTTAAGCTCGACAATCCAACCC |
| **AtKARI (SGNU440331) FWD** | CGGCGGCTACGGTCACTTCTTT |
| **AtKARI (SGNU440331) REV** | CGGCTTAAGCGATGGGGACAAA |
| **Asn2 (SGNU430910) FWD** | AACTCTCAGGCCAAGCGTTCCC |
| **Asn2 (SGNU430910) REV** | TGCCAACCGTTGATGAGCAAGA |
| **GHmetT (SGNU422294) FWD** | GGTTGGATCCTGCAAAATGGGG |
| **GHmetT (SGNU422294) REV** | TCCACCGTGGGGAAGATCAAGG |
| **EF-1α-F SGN-U446573** | GATTGGTGGTATTGGTACTGTC |
| **EF-1α-R, SGN-U446573** | AGCTTCGTGGTGCATCTC |
| **Actin F SGN-U431117** | ACGCCAGTGGCCGTACAACA |
| **Actin R SGN-U431117** | ATCGCGGACAATTTCCCGTTC |
| **Ubi3 F GB:X58253** | GCCGACTACAACATCCAGAAGG |
| **Ubi3R GB:X58253** | TGCAACACAGCAAGCTTAACC |
| **TMV-CP F** | CCCACGACTGCCGAAACGTTAG |
| **TMV-CP R** | TGCAGGACCAGAGGTCCAAACC |
